# Identification of Keratinocyte Differentiation-Involved Genes for Metastatic Melanoma by Gene Expression Profiles

**DOI:** 10.1155/2021/9652768

**Published:** 2021-12-28

**Authors:** Kezhu Li, Shu Guo, Shuang Tong, Qiang Sun, Shifeng Jin, Bingran Qi, Yining Shao, Nan Xu

**Affiliations:** Department of Plastic Surgery, The First Hospital of China Medical University, Shenyang, 11000 Liaoning Province, China

## Abstract

**Background:**

Melanoma is the deadliest type of skin cancer. Until now, its pathological mechanisms, particularly the mechanism of metastasis, remain largely unknown. Our study on the identification of genes in association with metastasis for melanoma provides a novel understanding of melanoma.

**Methods:**

From the Gene Expression Omnibus (GEO) database, the gene expression microarray datasets GSE46517, GSE7553, and GSE8401 were downloaded. We made use of R aiming at analyzing the differentially expressed genes (DEGs) between metastatic and nonmetastatic melanoma. R was also used in differentially expressed miRNA (DEM) data mining from GSE18509, GSE19387, GSE24996, GSE34460, GSE35579, GSE36236, and GSE54492 datasets referring to Li's study. Based on the DEG and DEM data, we performed functional enrichment analysis through the application of the DAVID database. Furthermore, we constructed the protein-protein interaction (PPI) network and established functional modules by making use of the STRING database. Through making use of Cytoscape, the PPI results were visualized. We predicted the targets of the DEMs through applying TargetScan, miRanda, and PITA databases and identified the overlapping genes between DEGs and predicted targets, followed by the construction of DEM-DEG pair network. The expressions of these keratinocyte differentiation-involved genes in Module 1 were identified based on the data from TCGA.

**Results:**

239 DEGs were screened out in all 3 datasets, which were inclusive of 21 positively regulated genes and 218 negatively regulated genes. Based on these 239 DEGs, we finished constructing the PPI network which was formed from 225 nodes and 846 edges. We finished establishing 3 functional modules. And we analyzed 92 overlapping genes and 26 miRNA, including 11 upregulated genes targeted by 11 negatively regulated DEMs and 81 downregulated genes targeted by 15 positively regulated DEMs. As proof of the differential expression of metastasis-associated genes, eleven keratinocyte differentiation-involved genes, including LOR, EVPL, SPRR1A, FLG, SPRR1B, SPRR2B, TGM1, DSP, CSTA, CDSN, and IVL in Module 1, were obviously downregulated in metastatic melanoma tissue in comparison with primary melanoma tissue based on the data from TCGA.

**Conclusion:**

239 melanoma metastasis-associated genes and 26 differentially expressed miRNA were identified in our study. The keratinocyte differentiation-involved genes may take part in melanoma metastasis, providing a latent molecular mechanism for this disease.

## 1. Introduction

Melanoma is the cells' neoplasm, starting in skin cells, namely, melanocytes [1]. Environmental factors, such as ultraviolet light exposure, are regarded as the main reason causing melanoma [2]. This tumor is mainly in the skin or adjacent to the skin and spreads throughout the body [3], with a dramatic increase in global incidence in the last few decades [4]. Metastasis, the most important characteristic of malignant tumors, is the primary reason for deaths in related to melanoma [5]. What is more, for patients with advanced melanoma, there are still no satisfactory treatments, which involve complicated changes in multiple genes and signaling pathways. Thus, investigating the latent molecular mechanisms of metastasis in melanoma is of great significance.

Recently, microarray technology has a wide range of applications in studying gene alterations in cancer recurrence, metastasis, tumorigenesis, drug resistance and identification of biomarkers for tumor prognosis, and diagnosing and treating the tumor [6–8]. By making use of RNA-sequencing analysis on many genes, as reported, RNAs, which contain long noncoding RNAs (lncRNAs), messenger RNAs (mRNAs), and miRNAs, and proteins have a critical influence on progression, melanoma initiation, and recurrence. MicroRNA is a type of noncoding RNA with a length of 18-25 nucleotides, which serves as a posttranscriptional regulator [9]. miRNA plays a role in human diseases by binding to the target mRNA in the 3-untranslated region (3-UTR). Previous reports have shown that miR-182 inhibits the proliferation of malignant melanoma cells through RECK [10]. Androgen receptor (AR) promotes melanoma metastasis by altering miRNA-539-3p/USP13/MITF/AXL signal [11]. In our study, we gained 3 mRNA microarrays and 7 miRNA microarrays aiming at analyzing the DEGs and DEMs between primary melanoma and metastatic melanoma tissue samples. We applied functional enrichment and network analysis. The results demonstrated latent molecular mechanisms on metastatic melanoma.

## 2. Material and Methods

### 2.1. Data Collection

We gained the GSE46517, GSE7553, and GSE8401 gene expression profiles and GSE18509, GSE19387, GSE24996, GSE34460, GSE35579, GSE36236, and GSE54492 miRNA expression profile from Gene Expression Omnibus (GEO, https://www.ncbi.nlm.nih.gov/geo/) [12]. The GSE46517 dataset included 8 normal skin samples, 9 nevus samples, 73 metastatic melanoma samples, and 31 primary melanoma samples [13]. GSE7553 was made up of 87 samples, including 5 normal samples, 15 basal cell carcinoma samples, 16 primary melanoma samples, 11 squamous cell carcinoma samples, and 40 metastatic melanoma samples [14]. GSE8401 contained 83 samples, 52 metastatic melanoma samples, and 31 primary melanoma samples included [15]. For these datasets, we screened out and analyzed metastatic melanoma samples. And we retrieved primary melanoma samples as control. Meanwhile, we collected seven miRNA expression profiles in total, which were inclusive of 82 metastatic and 87 nonmetastatic samples [12].

### 2.2. Screening DEGs and DEMs

We conducted the DEG and DEM analysis through the application of the limma software package in Bioconductor package (http://www.bioconductor.org/packages/release/bioc/html/limma.html) [16] in R software. In DEG analysis, our cutoff value was *p* < 0.01 and |fold change (FC) | >1.5, and we made use of FDR < 0.05 in DEM analysis [12]. Unique DEGs and DEMs were selected.

### 2.3. GO and Pathway Analysis of DEGs

As a predominant bioinformatics initiative, Gene Ontology (GO: http://www.geneontology.org/) [17] contains the most annotations under three headings: molecular function (MF), biological processes (BP), and cellular component (CC), while we performed the Kyoto Encyclopedia of Genes and Genomes (KEGG: http://www.genome.ad.jp/KEGG) [18] pathway enrichment analysis for the investigation of the signaling pathways that were in association with the unique DEGs. We made GO and KEGG pathway analysis through the Database for Annotation Visualization and Integrated Discovery (DAVID: http://www.david.ncifcrf.gov/) for the identification of the DEGs' biological significance [19]. We regarded FDR < 0.01 and gene count > 2 as statistical significance.

### 2.4. Protein-Protein Interaction (PPI) Network Construction

We first mapped the DEGs to the Search Tool for the Retrieval of Interacting Genes (STRING) (http://www.cytoscape.org/) [20] aiming at assessing functional associations among them. Then, through making use of the Molecular Complex Detection (MCODE), an app of Cytoscape software, we identified the functional modules of the PPI network.

### 2.5. miRNA Target Prediction and DEM-DEG Network Construction

We gained the predicted targets of miRNAs from TargetScan (http://www.targetscan.org/vert_72/) [21], miRanda (http://www.microrna.org/microrna/home.do) [22], and PITA (http://genie.weizmann.ac.il/pubs/mir07/mir07_data.html) databases [23]. The target genes which were predicted in at least two datasets were selected for the construction of the DEM-DEG pair network.

### 2.6. Validation of Differential Expression of Metastasis-Associated Genes

UALCAN, a comprehensive web resource for analyzing cancer OMICS data (TCGA and MET500), was used to validate the expression of 11 metastasis-associated genes enriched in “keratinocyte differentiation” of Module 1. The result was performed in boxplots, and we regarded *p* value < 0.05 to have statistical significance.

## 3. Results

### 3.1. Identification of DEGs and DEMs

**Figure 1:**
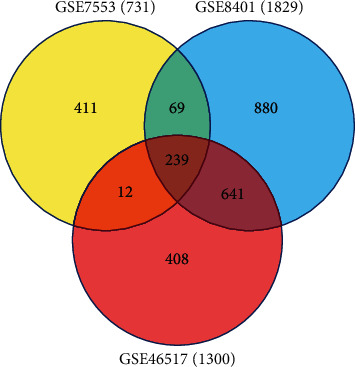
Identification of DEGs in mRNA expression profiling datasets GSE46517, GSE7553, and GSE8401.

For the identification of DEGs between primary melanoma samples and metastatic melanoma samples, through applying limma software package, we conducted a differential expression analysis. Totally, we identified 1300, 731, and 1829 DEGs to be significantly differentially expressed from GSE46517, GSE7553, and GSE8401, respectively. Finally, we screened out 239 genes in all 3 datasets, which were inclusive of 21 positively regulated genes and 218 negatively regulated genes in metastatic melanoma tissues in comparison with primary melanoma tissues (Figure 1). Meanwhile, we identified 63 DEMs from the seven miRNA expression profiles, which were made up of 35 positively negatively regulated miRNAs and 28 negatively regulated miRNAs in metastatic melanoma tissues in comparison with primary melanoma tissues (Table S1).

### 3.2. Functional Enrichment Analysis

**Figure 2:**
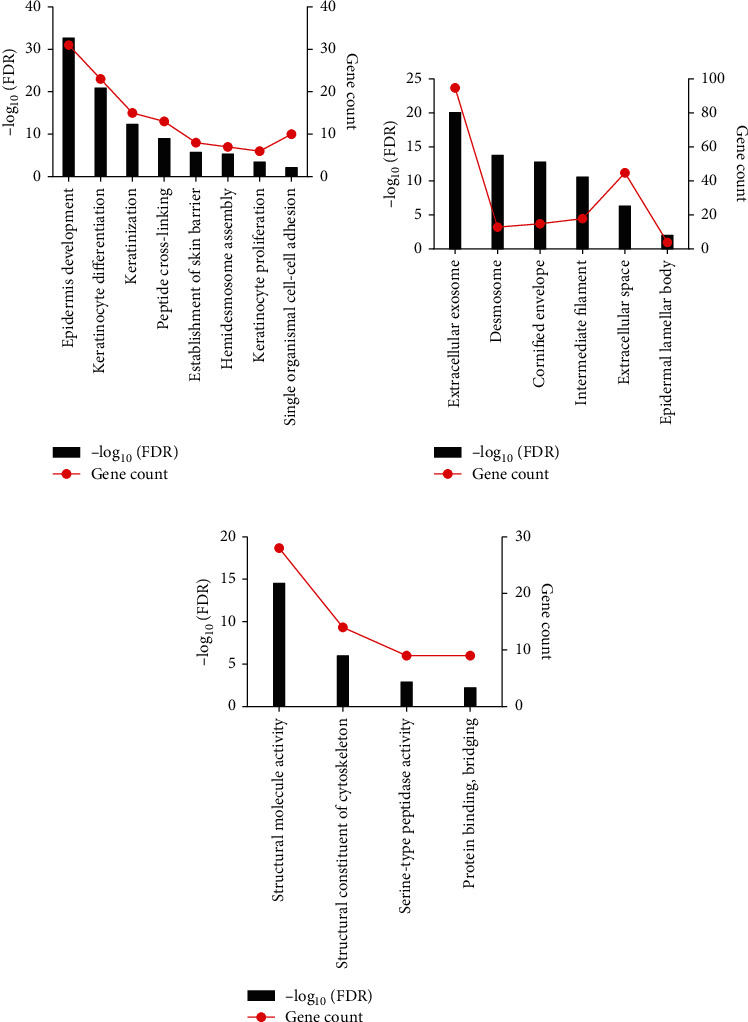
GO functions for the 239 DEGs: (a) enriched biological process of the negatively regulated genes; (b) enriched cellular component of negatively regulated genes; (c) enriched molecular function of negatively regulated genes.

We performed the functional enrichment analysis by making use of downregulated DEGs because the number of the DEGs (218/239) was large. We performed three categories of GO functional annotation analysis on DEGs, including BP, CC, and MF. The GO BP analysis results presented that negatively regulated DEGs were obviously abundant in epidermis development, keratinocyte differentiation, and keratinization (Figure 2(a), Table S2). The GO CC analysis showed that negatively regulated DEGs were abundant in extracellular exosome and desmosome (Figure 2(b), Table S2). The GO MF analysis results presented that negatively regulated DEGs were obviously abundant in structural molecule activity and structural constituent of cytoskeleton (Figure 2(c), Table S2). We only identified 2 KEGG pathways, considerably enhancing in the amoebiasis signaling pathway and arrhythmogenic right ventricular cardiomyopathy (ARVC) pathway (Table S2).

### 3.3. Construction and Analysis of PPI Network

**Figure 3:**
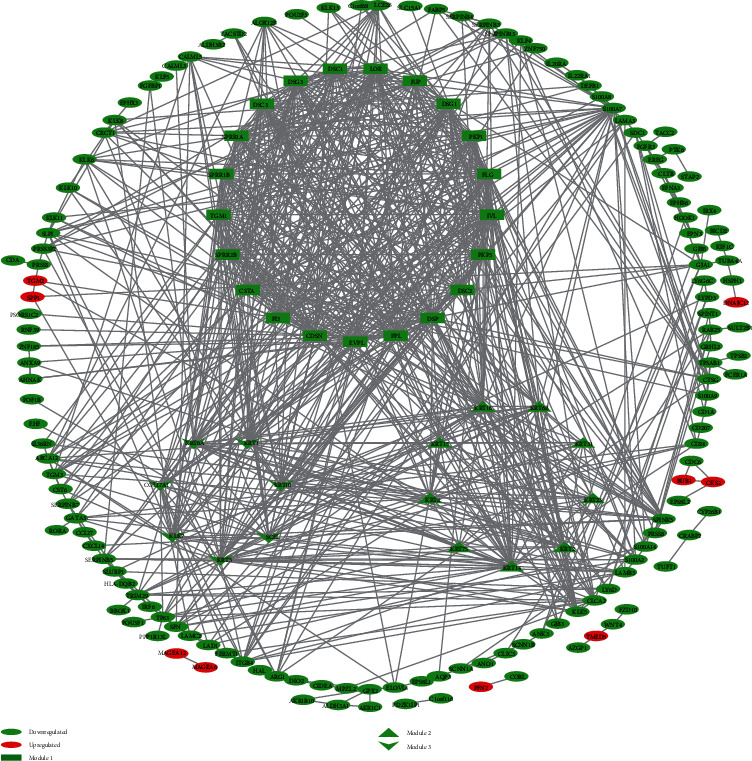
Protein-protein interaction network of DEGs. Green nodes stand for negatively regulated genes in melanoma metastasis tissue. Red nodes stand for positively regulated genes in melanoma metastasis tissue.

**Figure 4:**
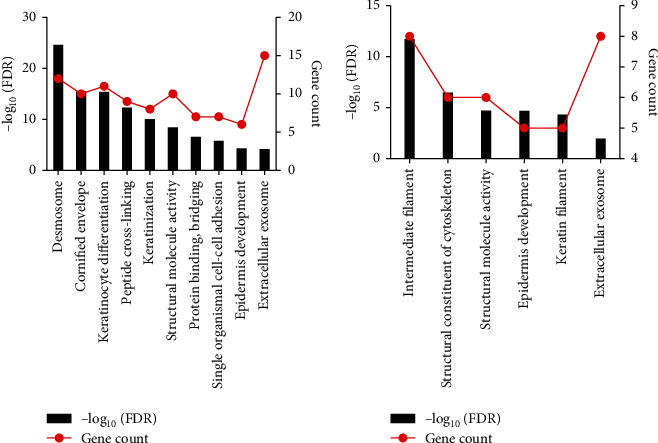
Significantly enriched GO terms for modules: (a) enriched BP, CC, and MF for Module 1; (b) enriched BP, CC, and MF for Module 2.

We performed the PPI network analysis of 239 DEGs through the STRING database, and 225 protein interactions with combined scores > 0.7 were identified (Figure 3). We identified three modules by making use of MCODE plugin, an app in Cytoscape. Module 1 was constructed with 21 nodes and 209 edges including DSC1, DSC2, and DSC3. Module 2 was made up of 9 nodes and 32 edges, which were inclusive of KRT2, KRT6B, and KRT8. Module 3 includes 7 nodes and 12 edges, which were made up of KRT1, KRT5, KRT6A, and so on. Furthermore, we performed DEGs' functional enrichment analysis in these 3 modules. The results demonstrated that Module 1 was enriched in the keratinocyte differentiation, extracellular exosome (Figure 4(a), Table S3), and so on in GO analysis. Module 2 was enriched in epidermis development, extracellular exosome (Figure 4(b), Table S3), and so on in GO analysis. Module 3 was enriched in epidermis development in GO analysis (Table S3). Unfortunately, we did not identify significant KEGG pathways in all three modules (FDR < 0.01).

### 3.4. Prediction of DEM Target Gene and Construction of DEM-DEG Network

We predicted 816 genes in total would be regulated by the 63 miRNAs identified above through making use of the TargetScan, PITA, and miRanda database. Among the 816 genes, 92 genes were overlapped with DEGs, being made up of 11 upregulated genes targeted by 11 negatively regulated DEMs and 81 genes downregulated genes targeted by 15 positively regulated DEMs. We presented the results in detail in Table S4.

**Figure 5:**
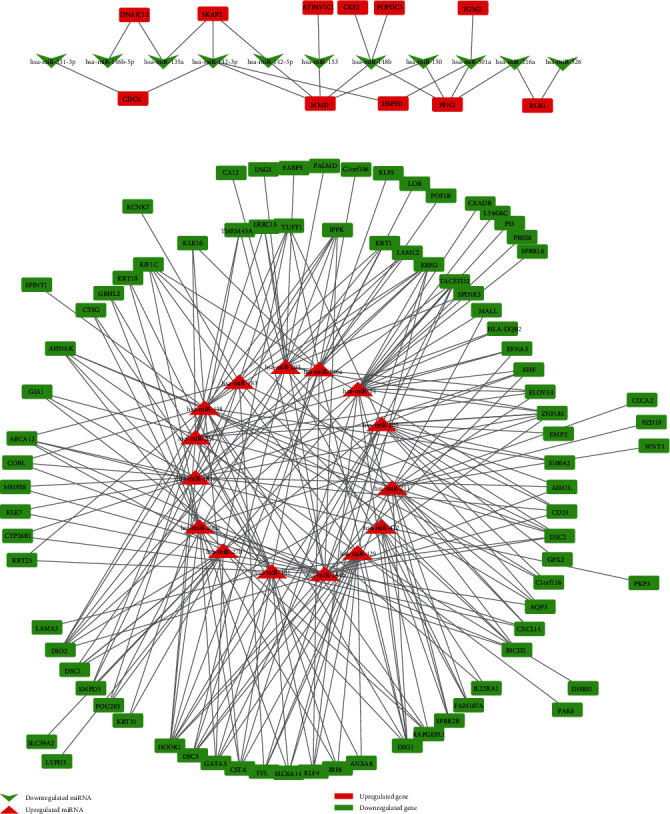
DEM-DEG pair network. Green nodes stand for negatively regulated genes or negatively regulated miRNA in melanoma metastasis tissue. Red nodes stand for positively regulated genes or positively regulated genes miRNA in melanoma metastasis tissue.

Subsequently, as presented in Figure 5, the DEM-DEG network was constructed by Cytoscape software. We found that hsa-miR-181b [24], hsa-miR-211 [25, 26], and hsa-miR-24 [27, 28], which had the most potential target genes, had a paramount influence on melanoma progression and metastasis.

### 3.5. Validation of Differential Expression of Metastasis-Associated Genes

**Figure 6:**
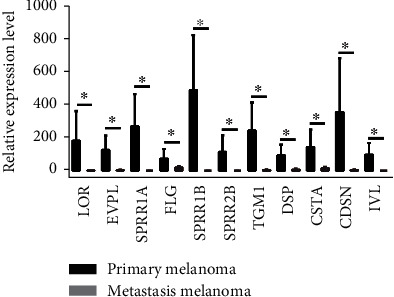
Validation of differential expression of metastasis-associated genes in Module 1. Expression level of LOR, EVPL, SPRR1A, FLG, SPRR1B, SPRR2B, TGM1, DSP, CSTA, CDSN, and IVL in metastasis melanoma (*n* = 368) and primary melanoma (*n* = 104) based on data from TCGA. Notes: ^∗^*p* < 0.01.

As shown in Table S3, Module 1 was obviously enriched in the keratinocyte differentiation in GO analysis. Until now, there were no detailed studies on keratinocyte differentiation in melanoma metastasis. As a result, we evaluated the expression of 11 enriched genes in keratinocyte differentiation through making use of the UALCAN database, including LOR, EVPL, SPRR1A, FLG, SPRR1B, SPRR2B, TGM1, DSP, CSTA, CDSN, and IVL (Figure 6). The analytic results demonstrated that all of the 11 genes were significantly downregulated in metastatic melanoma tissue in comparison to primary melanoma tissue. Some of the genes, such as LOR, SPRR1B, and IVL, were even undetectable in metastatic melanoma tissue.

## 4. Discussion

Poor outcome of melanoma is primarily caused by metastasis. The reason for melanoma metastasis is still poorly understood to date. The more we know about the pathogenesis of metastasis, the better the drug can be developed and this disease can be treated. More and more evidence demonstrated that gene expression profiling analysis is of great use for humans to study the cancer progression and metastasis. We have also identified an abundance of genes involved in many crucial cellular pathways of cancer progression and found their expression in melanoma was aberrant.

The value of studies on metastasis-associated genes for melanoma by gene expression profiling is relatively limited to date. In this research, we collected gene expression profiling datasets of melanoma and made a systemic meta-analysis to retrieve genes in association with metastasis. We identified 239 DEGs in total. Many of them were known to take part in various cancer types. For example, DIO2 was underexpressed in nearly all papillary thyroid carcinomas and known as a latent target for thyroid tumor treatment [29, 30]. FGFBP1 was identified as a prometastasis gene in HCC [31], whereas S100 family genes' expressions, particularly S100A7, were high in primary melanoma samples but low in metastatic melanoma. S100A7's expression was in relation to tumor invasion, and it may enhance melanoma's early diagnosis [32–34]. Additionally, it has been suggested that many predicted targets of DEGs were associated with cancer development. Interestingly, we found that most of the DEGs were downregulated (218/239). We identified DEGs' expression in TCGA database, and the results were the same as our analysis. But the reason is unknown.

**Figure 7:**
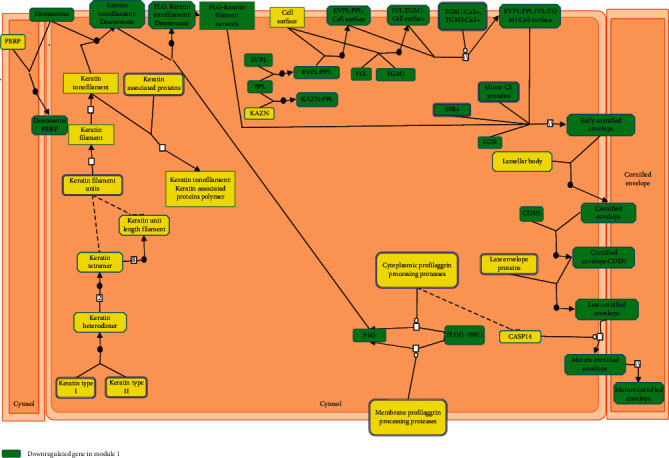
The reactome pathway diagram of the function of DEGs in Module 1 in keratinocyte differentiation.

Furthermore, we finished constructing the PPI network based on the DEGs. What is more, GO and KEGG pathway enrichment analyses were to further interpret their biological functions. For example, we found that 11 DEGs were enriched in keratinocyte differentiation GO term and close relation to melanoma metastasis (Figure 7). We found LOR, EVPL, SPRR1A, FLG, DSP, and CSTA had been reported in previous studies on melanoma [14, 27, 35–39]. But the report of SPRR1B, SPRR2B, TGM1, CDSN, and IVL in melanoma was a gap. Overall, this systematic meta-analysis of gene profiling was a step forward in the investigation of the mechanisms underlying melanoma's metastasis, and the role of SPRR1B, SPRR2B, TGM1, CDSN, and IVL in keratinocyte differentiation GO term needs to be further studied.

This study has some limitations. First, the expression levels of genes related to keratinocyte differentiation were not verified by qRT-PCR. Secondly, the role of miRNA-mRNA in melanoma metastasis needs further experimental verification. In future research, we will carry out these explorations.

To sum up, we finished the identification of many biological genes, which may take part in melanoma's metastasis, by collecting the gene expression datasets from the open database. This work identified keratinocyte differentiation-involved genes' role in melanoma metastasis for the first time, offering additional insights into this disease's complicated process.

## Data Availability

All data analyzed during this study are obtained from published article or are available from the corresponding author on reasonable request.

## Supplementary Materials

Supplementary 1 Table S1: differentially expressed miRNAs (DEMs) in melanoma.

Supplementary 2 Table S2: significantly enriched GO terms and KEGG pathways (downregulated).

Supplementary 3 Table S3: significantly enriched GO terms for modules.

Supplementary 4 Table S4: prediction of target genes for DEMs.
