# Supplementary material for: Identification of Keratinocyte Differentiation-Involved Genes for Metastatic Melanoma by Gene Expression Profiles
Source: Comput Math Methods Med. 2021 Dec 28;2021:9652768. doi: 10.1155/2021/9652768 (PMC8728391; doi:10.1155/2021/9652768)
Supplement: Supplementary 1 — Table S1: differentially expressed miRNAs (DEMs) in melanoma. [file 9652768.f1.docx]

| Table S1 Differentially expressed miRNAs (DEMs) in melanoma | | | | |
| --- | --- | --- | --- | --- |
| MicroRNA | logFC | AveExpr | *P* | Adjusted *P* |
| hsa‑mir‑205 | 3.84074 | 3.24142 | 7.59E‑22 | 7.94E‑19 |
| hsa‑mir‑203 | 3.27052 | 5.96689 | 3.29E‑16 | 1.72E‑13 |
| hsa‑mir‑508 | 1.55319 | 8.35084 | 1.09E‑05 | 6.73E‑04 |
| hsa‑mir‑514‑1 | 1.52272 | 5.78308 | 6.32E‑06 | 4.72E‑04 |
| hsa‑mir‑509‑1 | 1.44397 | 6.10834 | 1.67E‑05 | 7.61E‑04 |
| hsa‑mir‑506 | 1.35088 | 4.45629 | 1.28E‑05 | 7.03E‑04 |
| hsa‑mir‑211 | 1.33458 | 6.55326 | 2.07E‑04 | 6.00E‑03 |
| hsa‑mir‑200a | 1.30001 | 2.2969 | 8.35E‑13 | 2.91E‑10 |
| hsa‑mir‑141 | 1.2695 | 2.03624 | 1.62E‑09 | 2.82E‑07 |
| hsa‑mir‑513c | 1.12724 | 3.19562 | 1.25E‑05 | 7.03E‑04 |
| hsa‑mir‑891a | 1.04187 | 2.75798 | 2.31E‑04 | 6.38E‑03 |
| hsa‑mir‑514b | 0.93581 | 2.59849 | 1.02E‑04 | 3.54E‑03 |
| hsa‑mir‑513b | 0.85382 | 1.94141 | 9.66E‑06 | 6.31E‑04 |
| hsa‑mir‑513a‑1 | 0.8301 | 1.90886 | 1.42E‑05 | 7.17E‑04 |
| hsa‑mir‑944 | 0.77757 | 1.04188 | 1.08E‑06 | 1.03E‑04 |
| hsa‑mir‑510 | 0.63425 | 1.55961 | 2.04E‑04 | 6.00E‑03 |
| hsa‑mir‑507 | 0.621863 | 1.70567 | 4.91E‑04 | 1.14E‑02 |
| hsa‑mir‑183 | 0.54496 | 5.9569 | 3.02E‑03 | 4.41E‑02 |
| hsa‑mir‑224 | 0.51577 | 2.71914 | 2.54E‑04 | 6.80E‑03 |
| hsa‑mir‑429 | 0.50968 | 0.56736 | 1.01E‑07 | 1.17E‑05 |
| hsa‑mir‑146a | 0.5088 | 7.55563 | 1.66E‑03 | 2.75E‑02 |
| hsa‑mir‑27b | 0.5081 | 6.70707 | 2.52E‑05 | 1.02E‑03 |
| hsa‑mir‑138‑1 | 0.46358 | 2.74963 | 6.40E‑04 | 1.36E‑02 |
| hsa‑mir‑412 | 0.45386 | 1.42936 | 2.94E‑03 | 4.39E‑02 |
| hsa‑mir‑452 | 0.42736 | 3.19012 | 1.07E‑03 | 1.89E‑02 |
| hsa‑mir‑138‑2 | 0.42166 | 2.22355 | 6.58E‑04 | 1.36E‑02 |
| hsa‑mir‑23b | 0.41741 | 6.85683 | 1.45E‑05 | 7.17E‑04 |
| hsa‑mir‑181a‑2 | 0.37022 | 8.27776 | 3.04E‑03 | 4.41E‑02 |
| hsa‑mir‑181b‑2 | 0.36869 | 2.94543 | 3.34E‑03 | 4.65E‑02 |
| hsa‑mir‑24‑1 | 0.36819 | 2.55684 | 3.74E‑04 | 8.89E‑03 |
| hsa‑mir‑892a | 0.34883 | 0.45104 | 3.44E‑03 | 4.67E‑02 |
| hsa‑mir‑892b | 0.24103 | 0.18846 | 1.50E‑03 | 2.57E‑02 |
| hsa‑mir‑2115 | 0.21607 | 0.29474 | 1.30E‑04 | 4.37E‑03 |
| hsa‑mir‑573 | 0.19097 | 0.30461 | 2.14E‑03 | 3.35E‑02 |
| hsa‑mir‑23c | 0.18185 | 0.33044 | 6.67E‑04 | 1.36E‑02 |
| hsa‑mir‑663b | −0.03914 | 0.01899 | 1.15E‑03 | 2.01E‑02 |
| hsa‑mir‑548e | −0.18424 | 0.42785 | 8.56E‑04 | 1.69E‑02 |
| hsa‑mir‑548b | −0.18499 | 0.38084 | 3.43E‑03 | 4.67E‑02 |
| hsa‑mir‑216a | −0.21139 | 0.23894 | 0.00018 | 5.69E‑03 |
| hsa‑mir‑1224 | −0.21655 | 0.23499 | 0.000539 | 1.20E‑02 |
| hsa‑mir‑148b | −0.22071 | 5.67531 | 2.90E‑03 | 4.39E‑02 |
| hsa‑mir‑218‑1 | −0.24822 | 0.67965 | 3.63E‑03 | 4.86E‑02 |
| hsa‑mir‑135a‑1 | −0.28062 | 0.25828 | 1.41E‑04 | 4.59E‑03 |
| hsa‑mir‑1229 | −0.29592 | 0.90717 | 1.53E‑03 | 2.58E‑02 |
| hsa‑mir‑331 | −0.30701 | 3.22686 | 1.70E‑03 | 2.75E‑02 |
| hsa‑mir‑301a | −0.32842 | 2.17655 | 3.22E‑03 | 4.62E‑02 |
| hsa‑mir‑505 | −0.33969 | 4.04555 | 9.65E‑04 | 1.76E‑02 |
| hsa‑mir‑215 | −0.37497 | 1.44599 | 9.66E‑04 | 1.76E‑02 |
| hsa‑mir‑504 | −0.37641 | 0.94157 | 9.65E‑04 | 1.76E‑02 |
| hsa‑mir‑146b | −0.41183 | 6.82078 | 3.04E‑04 | 7.75E‑03 |
| hsa‑mir‑642a | −0.42094 | 1.53763 | 1.83E‑03 | 2.90E‑02 |
| hsa‑mir‑218‑2 | −0.45173 | 3.18601 | 1.96E‑04 | 6.00E‑03 |
| hsa‑mir‑125b‑1 | −0.45657 | 6.93287 | 6.77E‑04 | 1.36E‑02 |
| hsa‑mir‑326 | −0.47963 | 2.38721 | 5.62E‑04 | 1.22E‑02 |
| hsa‑mir‑766 | −0.54186 | 3.06395 | 1.51E‑05 | 7.17E‑04 |
| hsa‑mir‑153‑2 | −0.57756 | 1.01633 | 6.56E‑07 | 6.86E‑05 |
| hsa‑mir‑342 | −0.58209 | 5.68141 | 2.83E‑05 | 1.10E‑03 |
| hsa‑mir‑29c | −0.62039 | 6.12865 | 5.82E‑05 | 2.10E‑03 |
| hsa‑mir‑625 | −0.62962 | 5.73921 | 9.40E‑09 | 1.40E‑06 |
| hsa‑mir‑100 | −0.66570 | 8.64832 | 2.32E‑04 | 6.38E‑03 |
| hsa‑mir‑150 | −0.69533 | 6.69923 | 3.30E‑03 | 4.65E‑02 |
| hsa‑mir‑142 | −0.73181 | 6.46511 | 3.02E‑04 | 7.75E‑03 |
| hsa‑mir‑675 | −1.01107 | 2.712853 | 7.66E‑08 | 1.00E‑05 |
