# Supplementary material for: Identification of Keratinocyte Differentiation-Involved Genes for Metastatic Melanoma by Gene Expression Profiles
Source: Comput Math Methods Med. 2021 Dec 28;2021:9652768. doi: 10.1155/2021/9652768 (PMC8728391; doi:10.1155/2021/9652768)
Supplement: Supplementary 2 — Table S2: significantly enriched GO terms and KEGG pathways (downregulated). [file 9652768.f2.docx]

| Table S2 Significantly enriched GO terms and KEGG pathways (downregulated) | | | | |
| --- | --- | --- | --- | --- |
| **Terms** | **Description** | **Count (%)** | **genes** | **FDR** |
| **GO BP** |  |  |  |  |
| GO:0008544 | epidermis development | 31(14.09%) | S100A7, CRABP2, CDSN, COL17A1, LAMB3, KRT5, POU2F3, SPRR2B, KRT2, ZNF750, CALML5, KLK7, KLK5, LCE2B, KRT31, GJB5, GRHL2, SCEL, C1ORF68, EVPL, LAMA3, CST6, KRT17, SPRR1A, KRT16, SPRR1B, KRT15, KRT14, DSP, LAMC2, FABP5 | 1.75E-33 |
| GO:0030216 | keratinocyte differentiation | 23(10.45%) | LOR, S100A7, TP63, LCE2B, KRT10, CDSN, SCEL, C1ORF68, EVPL, CRCT1, EREG, SPRR1A, IRF6, FLG, KRT16, SPRR1B, SPRR2B, TGM1, DSP, TGM3, POU3F1, CSTA, IVL | 1.04E-21 |
| GO:0031424 | keratinization | 15(6.82%) | LOR, LCE2B, SFN, EVPL, KRT17, SPRR1A, KRT16, SPRR1B, PPL, SPRR2B, TGM1, TGM3, KRT2, IVL, ABCA12 | 3.55E-13 |
| GO:0018149 | peptide cross-linking | 13(5.91%) | LOR, C1ORF68, EVPL, CRCT1, SPRR1A, SPRR1B, SPRR2B, TGM1, DSP, TGM3, LCE2B, CSTA, IVL | 8.07E-10 |
| GO:0061436 | establishment of skin barrier | 8(3.64%) | FLG, KRT16, CYP26B1, KRT1, ALOX12B, TP63, SFN, ABCA12 | 1.44E-06 |
| GO:0031581 | hemidesmosome assembly | 7(3.18%) | COL17A1, LAMB3, LAMA3, KRT5, KRT14, ITGB4, LAMC2 | 3.78E-06 |
| GO:0043616 | keratinocyte proliferation | 6(2.73%) | KLK8, EREG, IRF6, FERMT1, TP63, KRT2 | 2.75E-04 |
| GO:0016337 | single organismal cell-cell adhesion | 10(4.55%) | JUP, ANXA9, MPZL2, PKP1, PKP3, DSG1, DSP, CSTA, CD24, CDSN | 5.42E-03 |
| **GO CC** |  |  |  |  |
| GO:0070062 | extracellular exosome | 95(43.18%) | CKMT1B, SLURP1, S100A8, S100A7, ANO1, S100A9, CRABP2, GJA1, SULT2B1, LRRC15, CDSN, SPINK5, BBOX1, GPX2, AZGP1, WNT4, KLK11, RAB25, AHNAK, AKR1C1, KLK13, EPN3, LAD1, SPINT1, KRT10, CYB5A, GLTP, SCEL, JUP, SDC1, EVPL, KRT17, CLIC3, CST6, SERPINB5, KRT16, ANXA8L1, TACSTD2, KRT15, AKR1B10, KRT14, DSP, SLPI, TUBA4A, SERPINB4, SERPINB3, CSTA, EPS8L2, EPS8L1, CTSG, KRT6C, KRT6A, KRT6B, ITGB4, SFN, PRSS8, ARG1, ADIRF, ANXA9, KRT5, CALML3, PPL, KRT8, PRSS3, TGM1, KRT1, KRT2, TGM3, SERPINB13, CALML5, SCNN1B, SCNN1A, IVL, DEFB1, S100P, SLC6A14, LGALS7, KRT31, S100A14, PDZK1IP1, C1ORF68, KRT75, LGALS7B, CKMT1A, C1ORF116, LAMA3, PKP1, DSG3, IRF6, SPRR1B, PI3, DSG1, DSC2, DSC1, FABP5 | 6.60E-21 |
| GO:0030057 | desmosome | 13(5.91%) | CDSN, JUP, EVPL, PKP1, DSG3, PKP3, PPL, DSG1, DSC3, DSP, POF1B, DSC2, DSC1 | 1.30E-14 |
| GO:0001533 | cornified envelope | 15(6.82%) | LOR, C1ORF68, EVPL, CRCT1, CST6, SPRR1A, SPRR1B, SPRR2B, TGM1, DSP, LCE2B, CSTA, CDSN, IVL, SCEL | 1.33E-13 |
| GO:0005882 | intermediate filament | 18(8.18%) | KRT6C, KRT6A, KRT31, GJA1, KRT10, KRT23, JUP, KRT75, PKP1, KRT5, KRT17, FLG, KRT16, KRT15, KRT8, KRT14, DSP, KRT2 | 1.98E-11 |
| GO:0005615 | extracellular space | 45(20.45%) | LYPD3, SLURP1, S100A8, S100A9, SFN, CXADR, CCL27, ALDH3A1, IL37, PRSS8, ARG1, AZGP1, WNT4, PRSS2, PRSS3, KRT1, KRT2, KLK11, SERPINB13, DEFB1, FGFBP1, KLK13, KLK6, KLK7, KLK8, KLK5, LGALS7, SPINT1, KRT31, KRT10, IL36RN, LGALS7B, EREG, CST6, CXCL14, SERPINB5, TACSTD2, SERPINB7, SLPI, LAMC2, SERPINB4, CSTA, SERPINB3, TPSAB1, CTSG | 3.70E-07 |
| GO:0097209 | epidermal lamellar body | 4(1.82%) | KLK7, KLK5, SPINK5, ABCA12 | 7.52E-03 |
| **GO MF** |  |  |  |  |
| GO:0005198 | structural molecule activity | 28(12.73%) | LOR, KRT6C, KRT6A, CLTB, KRT23, LAMB3, KRT5, KRT8, SPRR2B, KRT1, IVL, LAD1, LCE2B, JUP, C1ORF68, KRT75, EVPL, CRCT1, LAMA3, KRT17, SPRR1A, FLG, KRT16, SPRR1B, KRT15, KRT14, DSP, CSTA | 2.70E-15 |
| GO:0005200 | structural constituent of cytoskeleton | 14(6.36%) | LOR, KRT6A, KRT6B, KRT31, KRT5, KRT17, ANK3, KRT16, KRT15, PPL, KRT14, KRT2, TUBA4A, DSP | 9.61E-07 |
| GO:0008236 | serine-type peptidase activity | 9(4.09%) | PRSS8, KLK7, PRSS2, KLK5, PRSS3, KLK11, KLK10, TPSB2, TPSAB1 | 1.13E-03 |
| GO:0030674 | protein binding, bridging | 9(4.09%) | LOR, CNKSR1, EVPL, SPRR1A, ANK3, SPRR1B, DSP, CSTA, IVL | 5.31E-03 |
| **KEGG pathway** |  |  |  |  |
| hsa05146 | Amoebiasis | 8(3.64%) | ARG1, LAMB3, LAMA3, SERPINB4, LAMC2, SERPINB13, SERPINB3, CTSG | 1.30E-04 |
| hsa05412 | Arrhythmogenic right ventricular cardiomyopathy (ARVC) | 5(2.27%) | JUP, ITGB4, DSC2, GJA1, DSP | 5.67E-03 |
