# Supplementary material for: Identification of Keratinocyte Differentiation-Involved Genes for Metastatic Melanoma by Gene Expression Profiles
Source: Comput Math Methods Med. 2021 Dec 28;2021:9652768. doi: 10.1155/2021/9652768 (PMC8728391; doi:10.1155/2021/9652768)
Supplement: Supplementary 3 — Table S3: significantly enriched GO terms for modules. [file 9652768.f3.docx]

| Table S3 Significantly enriched GO terms for modules | | | | |
| --- | --- | --- | --- | --- |
| **Terms** | **Description** | **Count (%)** | **genes** | **FDR** |
| **Module 1** |  |  |  |  |
| GO:0030216 | keratinocyte differentiation | 11(52.38%) | LOR, EVPL, SPRR1A, FLG, SPRR1B, SPRR2B, TGM1, DSP, CSTA, CDSN, IVL | 3.40E-16 |
| GO:0018149 | peptide cross-linking | 9(42.86%) | LOR, EVPL, SPRR1A, SPRR1B, SPRR2B, TGM1, DSP, CSTA, IVL | 4.33E-13 |
| GO:0031424 | keratinization | 8(38.10%) | LOR, EVPL, SPRR1A, SPRR1B, PPL, SPRR2B, TGM1, IVL | 7.33E-11 |
| GO:0016337 | single organismal cell-cell adhesion | 7(33.33%) | JUP, PKP1, PKP3, DSG1, DSP, CSTA, CDSN | 1.45E-06 |
| GO:0008544 | epidermis development | 6(28.57%) | EVPL, SPRR1A, SPRR1B, SPRR2B, DSP, CDSN | 4.24E-05 |
| GO:0030057 | desmosome | 12(57.14%) | JUP, EVPL, PKP1, DSG3, PKP3, PPL, DSG1, DSC3, DSC2, DSP, DSC1, CDSN | 2.10E-25 |
| GO:0001533 | cornified envelope | 10(47.62%) | LOR, EVPL, SPRR1A, SPRR1B, SPRR2B, TGM1, DSP, CSTA, CDSN, IVL | 2.75E-16 |
| GO:0070062 | extracellular exosome | 15(71.43%) | CDSN, JUP, EVPL, PKP1, DSG3, SPRR1B, PPL, TGM1, PI3, DSG1, DSC2, DSP, DSC1, CSTA, IVL | 5.96E-05 |
| GO:0005198 | structural molecule activity | 10(47.62%) | LOR, JUP, EVPL, SPRR1A, FLG, SPRR1B, SPRR2B, DSP, CSTA, IVL | 3.30E-09 |
| GO:0030674 | protein binding, bridging | 7(33.33%) | LOR, EVPL, SPRR1A, SPRR1B, DSP, CSTA, IVL | 2.32E-07 |
| **Module 2** |  |  |  |  |
| GO:0008544 | epidermis development | 5(55.56%) | KRT16, KRT15, KRT14, KRT2, KRT31 | 1.85E-05 |
| GO:0005882 | intermediate filament | 8(88.89%) | KRT75, KRT16, KRT15, KRT8, KRT14, KRT2, KRT31, KRT23 | 1.74E-12 |
| GO:0045095 | keratin filament | 5(55.56%) | KRT75, KRT6B, KRT8, KRT14, KRT2 | 4.40E-05 |
| GO:0070062 | extracellular exosome | 8(88.89%) | KRT75, KRT6B, KRT16, KRT15, KRT8, KRT14, KRT2, KRT31 | 1.00E-02 |
| GO:0005200 | structural constituent of cytoskeleton | 6(66.67%) | KRT6B, KRT16, KRT15, KRT14, KRT2, KRT31 | 3.05E-07 |
| GO:0005198 | structural molecule activity | 6(66.67%) | KRT75, KRT16, KRT15, KRT8, KRT14, KRT23 | 1.79E-05 |
| **Module 3** |  |  |  |  |
| GO:0008544 | epidermis development | 4(57.14%) | KLK7, COL17A1, KRT5, SCEL | 1.94E-03 |
