# Supplementary material for: Identification of Keratinocyte Differentiation-Involved Genes for Metastatic Melanoma by Gene Expression Profiles
Source: Comput Math Methods Med. 2021 Dec 28;2021:9652768. doi: 10.1155/2021/9652768 (PMC8728391; doi:10.1155/2021/9652768)
Supplement: Supplementary 4 — Table S4: prediction of target genes for DEMs. [file 9652768.f4.docx]

| Table S4 Prediction of target genes for DEMs | |
| --- | --- |
| miRNA | Gene Symbol |
| hsa-miR-138 | AHNAK |
| hsa-miR-138 | AQP3 |
| hsa-miR-138 | C1orf116 |
| hsa-miR-138 | COBL |
| hsa-miR-138 | CSTA |
| hsa-miR-138 | DIO2 |
| hsa-miR-138 | DSC2 |
| hsa-miR-138 | DSG1 |
| hsa-miR-138 | EREG |
| hsa-miR-138 | IPPK |
| hsa-miR-138 | KLK10 |
| hsa-miR-138 | KRT1 |
| hsa-miR-138 | KRT23 |
| hsa-miR-138 | LAMC2 |
| hsa-miR-138 | POU2F3 |
| hsa-miR-138 | RAPGEFL1 |
| hsa-miR-138 | S100A2 |
| hsa-miR-138 | SMPD3 |
| hsa-miR-138 | SPINT1 |
| hsa-miR-141 | AHNAK |
| hsa-miR-141 | BICD2 |
| hsa-miR-141 | CSTA |
| hsa-miR-141 | CTSG |
| hsa-miR-141 | CXCL14 |
| hsa-miR-141 | DSC2 |
| hsa-miR-141 | GATA3 |
| hsa-miR-141 | GJA1 |
| hsa-miR-141 | GRHL2 |
| hsa-miR-141 | HOOK1 |
| hsa-miR-141 | IPPK |
| hsa-miR-141 | IRF6 |
| hsa-miR-141 | KIF1C |
| hsa-miR-141 | KLF4 |
| hsa-miR-141 | KRT10 |
| hsa-miR-141 | LAMA3 |
| hsa-miR-141 | RAPGEFL1 |
| hsa-miR-141 | SLC6A14 |
| hsa-miR-146a | C1orf106 |
| hsa-miR-146a | CD24 |
| hsa-miR-146a | DIO2 |
| hsa-miR-146a | DSC3 |
| hsa-miR-146a | DSG1 |
| hsa-miR-146a | EREG |
| hsa-miR-146a | HOOK1 |
| hsa-miR-146a | KIF1C |
| hsa-miR-146a | KLF4 |
| hsa-miR-146a | KLF5 |
| hsa-miR-146a | KLK10 |
| hsa-miR-146a | KRT1 |
| hsa-miR-146a | LAMC2 |
| hsa-miR-146a | LOR |
| hsa-miR-146a | POF1B |
| hsa-miR-146a | TUFT1 |
| hsa-miR-181a | ABCA12 |
| hsa-miR-181a | AIM1L |
| hsa-miR-181a | CD24 |
| hsa-miR-181a | COBL |
| hsa-miR-181a | CYP26B1 |
| hsa-miR-181a | DIO2 |
| hsa-miR-181a | EHF |
| hsa-miR-181a | EREG |
| hsa-miR-181a | GJA1 |
| hsa-miR-181a | HOOK1 |
| hsa-miR-181a | IPPK |
| hsa-miR-181a | KLK7 |
| hsa-miR-181a | MMP28 |
| hsa-miR-181a | TACSTD2 |
| hsa-miR-181a | TMEM45A |
| hsa-miR-181a | ZNF185 |
| hsa-miR-181b | ABCA12 |
| hsa-miR-181b | AIM1L |
| hsa-miR-181b | AQP3 |
| hsa-miR-181b | CD24 |
| hsa-miR-181b | COBL |
| hsa-miR-181b | CYP26B1 |
| hsa-miR-181b | DIO2 |
| hsa-miR-181b | DSC2 |
| hsa-miR-181b | EHF |
| hsa-miR-181b | EREG |
| hsa-miR-181b | HOOK1 |
| hsa-miR-181b | IPPK |
| hsa-miR-181b | IVL |
| hsa-miR-181b | KLK7 |
| hsa-miR-181b | KRT23 |
| hsa-miR-181b | LRRC15 |
| hsa-miR-181b | MMP28 |
| hsa-miR-181b | TACSTD2 |
| hsa-miR-181b | TMEM45A |
| hsa-miR-181b | ZNF185 |
| hsa-miR-183 | ABCA12 |
| hsa-miR-183 | BICD2 |
| hsa-miR-183 | DSC2 |
| hsa-miR-183 | IPPK |
| hsa-miR-183 | IVL |
| hsa-miR-183 | KCNK7 |
| hsa-miR-183 | KRT31 |
| hsa-miR-183 | SMPD3 |
| hsa-miR-183 | TUFT1 |
| hsa-miR-200a | AHNAK |
| hsa-miR-200a | BICD2 |
| hsa-miR-200a | CSTA |
| hsa-miR-200a | CTSG |
| hsa-miR-200a | CXCL14 |
| hsa-miR-200a | GATA3 |
| hsa-miR-200a | GRHL2 |
| hsa-miR-200a | HOOK1 |
| hsa-miR-200a | IRF6 |
| hsa-miR-200a | KIF1C |
| hsa-miR-200a | KRT10 |
| hsa-miR-200a | RAPGEFL1 |
| hsa-miR-200a | SLC6A14 |
| hsa-miR-200a | ZNF185 |
| hsa-miR-203 | CA12 |
| hsa-miR-203 | CXCL14 |
| hsa-miR-203 | DSC1 |
| hsa-miR-203 | DSC3 |
| hsa-miR-203 | DSG3 |
| hsa-miR-203 | ELOVL4 |
| hsa-miR-203 | EREG |
| hsa-miR-203 | FABP5 |
| hsa-miR-203 | FAM107A |
| hsa-miR-203 | IPPK |
| hsa-miR-203 | KRT1 |
| hsa-miR-203 | LAMC2 |
| hsa-miR-203 | LRRC15 |
| hsa-miR-203 | PALMD |
| hsa-miR-203 | POU2F3 |
| hsa-miR-203 | SPINK5 |
| hsa-miR-203 | ZNF185 |
| hsa-miR-205 | AIM1L |
| hsa-miR-205 | AQP3 |
| hsa-miR-205 | C1orf116 |
| hsa-miR-205 | CD24 |
| hsa-miR-205 | DSC1 |
| hsa-miR-205 | DSC2 |
| hsa-miR-205 | DSC3 |
| hsa-miR-205 | EHF |
| hsa-miR-205 | EMP2 |
| hsa-miR-205 | GATA3 |
| hsa-miR-205 | GPX2 |
| hsa-miR-205 | HOOK1 |
| hsa-miR-205 | S100A2 |
| hsa-miR-205 | SLC6A14 |
| hsa-miR-205 | TACSTD2 |
| hsa-miR-205 | TUFT1 |
| hsa-miR-205 | ZNF185 |
| hsa-miR-211 | AHNAK |
| hsa-miR-211 | AQP3 |
| hsa-miR-211 | BICD2 |
| hsa-miR-211 | C1orf116 |
| hsa-miR-211 | CLCA2 |
| hsa-miR-211 | CXCL14 |
| hsa-miR-211 | DSC1 |
| hsa-miR-211 | DSC2 |
| hsa-miR-211 | DSC3 |
| hsa-miR-211 | DSG1 |
| hsa-miR-211 | EFNA3 |
| hsa-miR-211 | ELOVL4 |
| hsa-miR-211 | FAM107A |
| hsa-miR-211 | FZD10 |
| hsa-miR-211 | GJA1 |
| hsa-miR-211 | IVL |
| hsa-miR-211 | KIF1C |
| hsa-miR-211 | MALL |
| hsa-miR-211 | SPRR2B |
| hsa-miR-211 | WNT4 |
| hsa-miR-224 | ANXA8 |
| hsa-miR-224 | DIO2 |
| hsa-miR-224 | EFNA3 |
| hsa-miR-224 | ELOVL4 |
| hsa-miR-224 | HLA-DQB2 |
| hsa-miR-224 | HOOK1 |
| hsa-miR-224 | IL22RA1 |
| hsa-miR-224 | KLK10 |
| hsa-miR-224 | LRRC15 |
| hsa-miR-224 | TMEM45A |
| hsa-miR-224 | TUFT1 |
| hsa-miR-224 | ZNF185 |
| hsa-miR-24 | CSTA |
| hsa-miR-24 | CXADR |
| hsa-miR-24 | DIO2 |
| hsa-miR-24 | DSG1 |
| hsa-miR-24 | EFNA3 |
| hsa-miR-24 | EHF |
| hsa-miR-24 | ELOVL4 |
| hsa-miR-24 | EREG |
| hsa-miR-24 | HLA-DQB2 |
| hsa-miR-24 | KLF4 |
| hsa-miR-24 | LY6G6C |
| hsa-miR-24 | MALL |
| hsa-miR-24 | PI3 |
| hsa-miR-24 | PRSS8 |
| hsa-miR-24 | SPINK5 |
| hsa-miR-24 | SPRR1A |
| hsa-miR-24 | SPRR2B |
| hsa-miR-24 | TACSTD2 |
| hsa-miR-24 | ZNF185 |
| hsa-miR-27b | ABCA12 |
| hsa-miR-27b | DIO2 |
| hsa-miR-27b | DSC1 |
| hsa-miR-27b | DSG1 |
| hsa-miR-27b | GATA3 |
| hsa-miR-27b | HOOK1 |
| hsa-miR-27b | KRT31 |
| hsa-miR-27b | LYPD3 |
| hsa-miR-27b | POU2F3 |
| hsa-miR-27b | SLC39A2 |
| hsa-miR-27b | SMPD3 |
| hsa-miR-27b | TACSTD2 |
| hsa-miR-27b | TUFT1 |
| hsa-miR-412 | ABCA12 |
| hsa-miR-412 | BICD2 |
| hsa-miR-412 | DSG1 |
| hsa-miR-412 | FAM107A |
| hsa-miR-412 | IL22RA1 |
| hsa-miR-412 | PKP3 |
| hsa-miR-412 | RAPGEFL1 |
| hsa-miR-412 | SPRR2B |
| hsa-miR-429 | AHNAK |
| hsa-miR-429 | ANXA8 |
| hsa-miR-429 | CSTA |
| hsa-miR-429 | DHRS1 |
| hsa-miR-429 | DSC3 |
| hsa-miR-429 | DSG1 |
| hsa-miR-429 | EREG |
| hsa-miR-429 | GATA3 |
| hsa-miR-429 | HOOK1 |
| hsa-miR-429 | IRF6 |
| hsa-miR-429 | IVL |
| hsa-miR-429 | KIF1C |
| hsa-miR-429 | KLF4 |
| hsa-miR-429 | PAK6 |
| hsa-miR-429 | SLC6A14 |
| hsa-miR-135a | DNAJC12 |
| hsa-miR-135a | SKAP2 |
| hsa-miR-142-3p | CDC6 |
| hsa-miR-142-3p | HSPH1 |
| hsa-miR-142-3p | MMD |
| hsa-miR-142-3p | SKAP2 |
| hsa-miR-142-5p | MMD |
| hsa-miR-142-5p | SKAP2 |
| hsa-miR-146b-5p | DNAJC12 |
| hsa-miR-148b | CKS2 |
| hsa-miR-148b | MMD |
| hsa-miR-148b | PFN2 |
| hsa-miR-148b | POPDC3 |
| hsa-miR-150 | MMD |
| hsa-miR-150 | PFN2 |
| hsa-miR-153 | ATP6V1C1 |
| hsa-miR-153 | MMD |
| hsa-miR-216a | BUB1 |
| hsa-miR-216a | PFN2 |
| hsa-miR-301a | HSPH1 |
| hsa-miR-301a | PFN2 |
| hsa-miR-301a | TGM2 |
| hsa-miR-326 | BUB1 |
| hsa-miR-331-3p | CDC6 |
